# Trends and emerging research directions of sustainable aviation: A bibliometric analysis

**DOI:** 10.1016/j.heliyon.2024.e32306

**Published:** 2024-06-03

**Authors:** Fatma Cande Yaşar Dinçer, Gözde Yirmibeşoğlu, Yasemin Bilişli, Emel Arık, Hakkı Akgün

**Affiliations:** a Department of International Trade and Logistics, Faculty of Applied Sciences, Akdeniz University, 07070, Antalya, Türkiye; b Department of Office Services and Secretariat, Social Sciences Vocational School, Akdeniz University, 07070, Antalya, Türkiye; c Department of Journalism, Faculty of Communication, Akdeniz University, 07070, Antalya, Türkiye; d Department of Journalism, Faculty of Communication, Suleyman Demirel University, 32260, Isparta, Türkiye

**Keywords:** Aviation, Sustainability, Sustainable aviation, Bibliometric analysis

## Abstract

This study aims to conduct a bibliometric analysis to determine trends and emerging research directions of sustainable aviation between 2001 and 2023. 726 studies indexed in the Web of Science were examined through VOSviewer software. Science mapping and performance analyses were implemented to demonstrate a systematic quantitative review and the characteristics of the research area. Moreover, by using co-occurrence of keywords, citation, bibliographic coupling, co-authorship, and co-citation analyses, the trends of the research area were revealed in detail. Findings indicated that the publications on sustainable aviation literature were mainly conducted between 2020 and 2023. Research areas of the publications were mainly on “engineering” and “energy fuels”. In terms of number of the publications, “International Journal of Sustainable Aviation Fuel” was the most productive source and Heyne was the most productive author. Co-occurrence analysis demonstrated that “sustainable aviation fuel” was the most frequently used keyword. Furthermore, sustainable aviation research has shifted in focus toward more challenging and technology-oriented research over time. Citation analysis indicated that the most cited author was Heyne, the most cited study was Ma et al.’s study on “Aviation biofuel from renewable resources: routes, opportunities and challenges” and the most cited sources was “Energy”. Among countries, the U.S.A was the most cited country and Chinese Academy of Sciences was the most cited organization. Bibliographic analysis showed that Heyne was the author with the highest connection strength. Co-authorship analysis demonstrated that Washington State University was the most collaborative organization. Finally, co-citation analysis of cited references indicated that fundamental subjects and related references were mainly sustainable aviation fuel, production of sustainable aviation fuel and its use in aviation studies. It is anticipated that results of this study would contribute to sustainable aviation research and ensure guidance and new perspectives for future research topics and directions on sustainable aviation.

## 1 Introduction

Aviation industry has a remarkable impact on air quality and climate change. Upon the triggering effect of globalization and technological developments, emissions from aviation industry have been increasing significantly since 1990 and are estimated to increase incrementally by the middle of the century [1]. As the world population continues to increase and countries have developed modern aviation networks, there is a growing number of passengers who use air transportation [2]. Moreover, the more demand for air travel increases, so does the demand for new aircrafts, employees and airports [3,4]. These leads to an increase in the negative effects of aviation on the environment. Growing negative effects of aviation industry on environment have revealed the need to take environmental aspects into account in aviation operations.

To reduce the effects of aviation on the environment, people have paid increasingly attention to sustainable aviation issues. “Sustainable Aviation is a long-term strategy to tackle the challenge of providing a cleaner, quieter and smarter future for the aviation industry” [5]. It aims both to ensure sustainability of development in aviation activities and to alleviate their detrimental impact [6]. According to Seyam et al. [7], the sustainable aviation issues have attracted the attention of academic and industrial research in order to determine and mitigate negative impacts of aviation on environment. Because of the dramatic effects of aviation on climate change, researchers have increasingly attached importance on sustainable aviation research on the related literature especially since the beginning of the 21st century. On the other hand, scientific research on sustainable aviation is growing but still developing. Moreover, considering the existing literature, deficiency in ones using bibliometric methods among sustainable aviation studies was explicit. Therefore, the aim of this study is to examine the trends and emerging research directions of sustainable aviation literature by using science mapping and performance analyses as a part of bibliometric analysis.

As a result of the screening in the bibliometric analysis, a limited number of review studies on sustainable aviation were determined. Among 726 studies, 61 were review studies and 3 were book reviews. Review studies on sustainable aviation have mostly focused on sustainable aviation fuel, production of sustainable aviation fuel and its use in aviation [[8], [9], [10], [11], [12], [13], [14], [15], [16], [17], [18]], aircraft technologies and designs within the context of sustainable aviation [[19], [20], [21], [22], [23], [24], [25]] and life-cycle assessment [26,27]. On the other hand, to the best of our knowledge, retrospective features and trends of sustainable aviation literature have not yet been analyzed systematically. For this reason, there is a need for an in-depth examination of the literature to demonstrate the trends of the research area and determine research themes. Moreover, in the existing sustainable aviation literature, there is a need to map the published studies in a systematic manner to examine current and historical trends and potential gaps for future studies. Therefore, the present study was conducted to fill this gap in the literature. During the data collection stage, Web of Science (WoS) was used as a database and the analyses were performed by using via VOSviewer 1.6.19 software. In the study, a comprehensive review of sustainable aviation field was made by running performance and science mapping analyses.

The study consisted of five sections. After introduction in section 1, the researchers examined the literature on sustainable aviation in section 2. Section 3 gives information on data collection and processing and conceptual framework of bibliometric analysis. Section 4 contains results about bibliometric analysis and discussion part. In order to present an illustrative outlook of the current sustainable aviation research, performance analysis was run. Therefore, an overview of years, numbers, research areas and sources of publications as well as top publishing researchers was carried out via performance analysis. Then, co-occurrence of author keywords, citation analysis of authors, documents and sources, most cited countries and organizations, bibliographic coupling analysis of authors, co-authorship analysis of organizations and co-citation analysis of cited references were examined by performance analysis and/or science mapping analysis. Lastly, in the conclusion section, the researchers discussed the key findings of the study, provided implications for theory and practices, underlined limitations of the study, and gave recommendations for future research.

## 2 Literature review

As the demand for air transportation and the number of aircraft used worldwide increase, air pollution also intensifies significantly. The United Nations Framework Convention on Climate Change declares that aviation industry is among top 10 polluters and “aviation emissions are 2.1 % of the global share, but when non-CO_2_ effects are included, aviation contributes an estimated 4.9 % to the global warming problem” [1]. According to Ritchie [28], global aviation, including domestic, international, passenger and freight transportation, accounts for 2.5 % of global CO_2_ emissions; however, its effect on total global warming has reached up to 3.5 %. Moreover, current estimations show that demand for air passenger journeys could exceed 10 billion by 2050 [29,30]. IATA member airlines took a decision based on the limits of the Paris Agreement suggesting not to exceed 1.5 °C in global warming and committed to achieve net zero carbon emissions from their operations by 2050 in order to cope with aviation industry's negative effects on environment and progress toward more sustainable aviation [30].

Negative effects of aviation sector on environment and growing number of people using air travel rise environmental concerns about detrimental repercussions of this industry on climate change and necessitates to take precautions about emissions and conduct research on sustainable aviation. For this reason, academic attention toward sustainable aviation topics has been increasing. According to Walker and Cook [31] “Much of the research associated with sustainable aviation has a positivistic focus on more efficient operations and technology”. As a result of the literature review, it was identified that the studies focused on different issues and methods related to sustainable aviation. The authors mainly examined sustainable aviation fuel, its production and its use in aviation [[8], [9], [10], [11], [12], [13], [14], [15], [16], [17], [18]], aircraft technologies and designs within the context of sustainable aviation [[19], [20], [21], [22], [23], [24], [25]] and life-cycle assessment [26,27] in their studies.

“Sustainable aviation fuel (SAF) is the main term used by the aviation industry to describe a non-conventional (fossil derived) aviation fuel” [32]. Studies in the related literature have mostly focused on sustainable aviation fuel, its production and its use in aviation. Murphy et al. [8] reviewed on “biomass production for sustainable aviation fuels” in their regional case study and discussed “a range of sustainability issues associated with biomass production”. Peters et al. [9] reviewed “current and emerging production technologies for biomass-derived sustainable aviation fuels”. The results of the studies have indicated the necessity of improving the capacities of existing technologies to reduce greenhouse gas emissions from the aviation industry through bio jet fuel as well as creating new, sustainable, high-volume routes for commercial use of bio jet fuel.

Okolie et al. [10] analyses production pathways of sustainable aviation fuel like “hydroprocessed esters and fatty acids (HEFA), gasification and Fischer–Tropsch Process (GFT), Alcohol to Jet (ATJ), direct sugar to hydrocarbon (DSHC), and fast pyrolysis (FP)” via multi-criteria decision framework. According to the results of the study, in which it was assumed that technological and environmental criteria had the same weighted factor, the sustainable aviation fuel production pathways were considered as HEFA > DSHC > FP > ATJ > GFT. Furthermore, Hari et al. [11] examined “aviation biofuel from renewable resources” and highlighted that the industrialization of alternative aviation fuels produced from renewable sources is crucial to deal with greenhouse gas emissions. Cruz et al. [12] reviewed production of “biofuels from oilseed using different thermochemical processes”. They concluded that oilseed fruits could potentially be used as biofuels; however, doing so requires thermochemical improvements and new technology.

Alternative fuel usage in aviation industry is very significant to reduce dependence on fossil fuels and to overcome the negative effects of greenhouse gas emissions [17]. According to Cabrera and de Sousa [18], alternative fuel usage in aviation has an important role in both achieving future emission targets and reducing dependence on fossil fuels. Moreover, Yusaf et al. [13]state that hydrogen, which is abundant, clean and does not create carbon emissions, is a suitable alternative fuel. “Hydrogen is an energy carrier that when produced with renewable energy sources can greatly improve production of drop-in sustainable aviation fuels and has the potential to evolve into a low carbon impact fuel for use with propulsion, flight and infrastructure technologies of the future” [33].

Undavalli et al. [16] assessed hydrogen as a “potential competitor for SAFs and a non-carbon fuel with the potential to reduce CO_2_ emissions by 100 %, is still subject to the design challenges of aircraft systems, fuel storage, higher costs associated with fuel production, and new systems development”. In their studies, Dincer and Acar [14] examined “potential use of hydrogen in aviation applications” and determined that hydro-based electrolysis was the most advantageable hydrogen production option. According to Degirmenci et al. [15] sustainable aviation fuels, which will reduce the environmental impacts of the aviation industry, are considered as an alternative to fossil fuels, and “hydrogen, a clean energy carrier, is regarded as the most promising of these fuels”.

In addition to production of sustainable aviation fuel and its use in aviation, the second issue mostly examined on sustainable aviation is aircraft technologies and designs. According to Gardi et al. [22], an ever-increasing demand for air transportation rises the awareness about and interests in economically viable and sustainable aviation practices, leading to significant improvements in design and operations of aircraft, airspace, and airport systems. When aircraft technologies and designs were took into consideration within the context of sustainable aviation literature, Gardi et al. [22] studied Multi-Objective Trajectory Optimization (MOTO) techniques for aircraft flight operations and emphasized that MOTO algorithms had a remarkable potential on real-time planning and re-planning of flight routes both economically and environmentally optimized. Brooker [19] examined and discussed design priorities of civil aircraft from economic and environmental dimensions. Wang [20] reviewed “high-order computational fluid dynamic tools for aircraft design” and pointed out the importance of how high-order methods affect “new generation design tools for aircraft and engines”. Abu Salem et al. [21] analyzed “hybrid-electric aircraft technologies and designs” via critical analysis. They underlined that the electrical energy used in aircraft must be produced without CO_2_ emissions and the use of renewable resources for electricity production should be maximized in order to provide a positive contribution on the global warming issue.

Ranasinghe et al. [23] studied “low-emission technologies for sustainable aviation” and addressed the significance and necessity of disruptive technological advances to ensure fuel efficiency and diminish the detrimental impact of aviation on environment. Bravo-Mosquera et al. [24] examined the current state-of-the-art technology in the design of future aircraft for civil aviation by conducting a literature review and mentioned about “design and development of several unconventional aircraft configurations”. Moreover, Zhang et al. [25] gave information about recent developments on control system design and energy management for electric aircraft propulsion systems as well as their challenges and technical barriers. According to these authors, more electrified aircraft propulsion technologies have emerged due to the concerns of sustainability in the aviation sector and they have become a very promising approach toward future sustainable and decarbonized aviation.

Apart from the topics of production of sustainable aviation fuel and its use in aviation and aircraft technologies and designs, another reviewed topic in the sustainable aviation literature was life-cycle analysis (or life-cycle assessment). According to Finnveden et al. [34], “Life Cycle Assessment is a tool to assess the environmental impacts and resources used throughout a product's life cycle, i.e., from raw material acquisition, via production and use phases, to waste management”. “Life-cycle assessments are playing an increasingly important role as a method for the aviation industry” to realize the goal of reducing the negative impacts of the industry on environment [26]. In their studies, Hu et al. [27] studied strategies of mitigating carbon emission in sustainable aviation industry through life cycle analysis and Delphi method by creating a timetable of the low carbon technologies. The results of their study indicated that the most important factors affecting carbon emissions in the aviation industry were demand, technological development, and alternative fuels. Keiser et al. [26] systematically reviewed life-cycle assessment in aviation industry and concentrated on the related literature except for sustainable aviation fuel studies. They proposed a conceptual framework for the application of life-cycle analysis in the aviation industry, a methodological specification of life-cycle analysis and an organizational research agenda for its operationalization in this industry.

We examined previous review studies investigating sustainable aviation from different aspects; however, they reviewed the topic from a limited perspective and were mostly qualitative and theoretical. Moreover, these studies examined relatively few publications with limited topics and lack a quantitative analysis on the sustainable aviation literature. Therefore, the aim of this study is to determine trends and emerging research directions of sustainable aviation by means of using bibliometric analysis.

## 3 Materials and methods

### 3.1 Data collection and processing

**Fig. 1:**
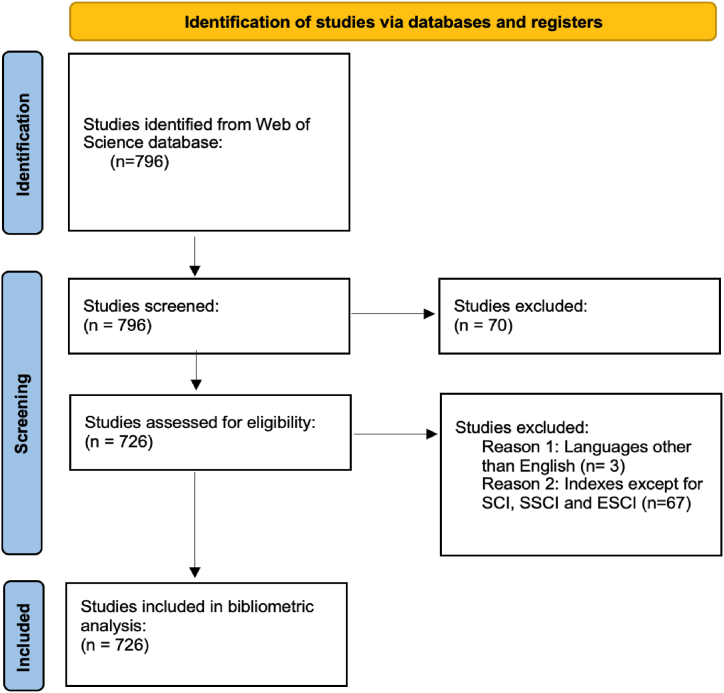
PRISMA Flow Diagram. Source: Author's work that was modified from Ref. [36].

According to van Eck and Waltman [35], VOSviewer offers the viewer bibliometric maps which can be viewed in full detail and “most of computer programs that are used for bibliometric mapping do not display such maps in a satisfactory way”. In this context, the bibliometric data used in this study was retrieved from Web of Science and the data were analyzed using VOSviewer 1.6.19 software. Moreover, PRISMA 2020 Flow Diagram was used to systematically address publications. PRISMA 2020 Flow Diagram which was revised and updated by Page et al. [36], to collect data for bibliometric analysis in systematic examination of publications. “The flow diagram depicts the flow of information through the different phases of a systematic review. It maps out the number of records identified, included and excluded, and the reasons for exclusions” [37]. Fig. 1 shows the PRISMA Flow Diagram for the data collection steps for bibliometric analysis.

As a consequence of the search for the keyword “sustainable aviation” in Web of Science database, 796 studies were accessed on November 19, 2023 for the period between 2001 and 2023. In the selection procedure, all types of publications in all areas were selected. Then, languages other than English and indexes except for SCI, SSCI and ESCI were chosen as the exclusion criteria. As a result of the screening, it was determined that 3 publications were written in languages other than English and 67 publications were conducted in the indexes except for SCI, SSCI and ESCI. Moreover, a bibliometric screening was conducted to identify irrelevant themes for exclusion but no irrelevant publications on sustainable aviation were found. Thus, the bibliometric screening of this study comprised 726 studies published between 2001 and 2023. Table 1 shows the distribution of publication types and indexes.

**Table 1:** Distribution of publication types and indexes.

Publication Types	Frequency (n)	Percent (%)
Article	638	87.878 %
Review Article	61	8.402 %
Proceeding Paper	40	0.55 %
Early Access	22	3.03 %
Editorial material	13	1.790 %
News Item	7	0.964 %
Book Review	3	0.4132 %
Meeting Abstract	3	0.4132 %
Correction	1	0.1377 %
Data Paper	1	0.1377 %
**Web of Science Index**		
SCI-Expanded	460	63.36 %
SSCI	69	9.504 %
ESCI	243	33.016 %

Note: Some publications may be included in more than one Web of Science Category.

After analyzing the studies, ten types of publication were determined. The publications were mostly articles (F = 638, P = 87.878) (Table 1). Other publications were review articles (F = 61, P = 8.402 %), proceeding papers (F = 40, P = 0.55 %), early accesses (F = 22, P = 3.03 %), editorial materials (F = 13, P = 1.790 %), news items (F = 7, P = 0.964), book reviews (F = 3, P = 0.4123), meeting abstracts (F = 3, P = 0.4132), correction (F = 1, P = 0.1377 %), and data paper (F = 1, P = 0.1377). Moreover, in terms of Web of Science Indexes, 460 publications (63.36 %) were indexed in SCI-Expanded, 69 (9.504 %) were indexed in SSCI and 243 (33.016 %) were indexed in ESCI.

### 3.2 Bibliometric analysis

In this study, bibliometric analysis was used to examine the literature on sustainable aviation. “Bibliometric analysis refers to the use of quantitative methods to analyze the existing body of literature” [38]. It is the numerical analysis of the publications produced by individuals or institutions in a certain subject, period, area and the relations between these publications. Nowadays, bibliometric analysis has been increasingly attracting the interest of scholars especially with the triggering effects of the rapid advancement of computers and the Internet [[39], [40], [41]].

Researchers define two types of bibliometric methods [42]; performance analysis and science mapping (or bibliometric mapping) analysis. Performance analysis is used to analyze the output of research on any scientific subject [43]. Science or bibliometric mapping, on the other hand, finds outs relationships between various parts of the research components and enables to assess documents, references, authors, organizations and productivities of countries [[44], [45], [46]]. Moreover, science mapping analysis “aims to visually present and display the conceptual, social or intellectual structures of scientific research, and the evolution, development, and dynamics of the research area” [47]. It is possible to benefit from science mapping analysis to indicate the cognitive design of the research area [42,43]. Input frequency (keywords, citations, documents etc.) is generally confined to a specific number to acquire explicit and accurate maps in science mapping analysis [48].

The first step of bibliometric analysis is to decide which data source is the most appropriate for the scope of the scientific field to make the correct analysis. According to Mulet-Forteza et al. [49], Web of Science “is considered as the most effective database”. In addition, it stands out as one of the mostly used and referred database in the disciplines of social sciences [50]. It contains almost all major research papers and ensures built-in analysis instruments to generate representative figures [51]. Moreover, searching outcomes can be analyzed in more details via VOSviewer software. According to Moral-Muñoz et al. [52], “VOSviewer has a fantastic visualization and is capable of loading and exporting information from many sources” while compared to other analysis tools. Providing a representative and informative view of the data, bibliometric mappings are very useful to graphically represent bibliographic materials [35]. They gather data and create maps based on co-authorship, co-occurrence of keywords, citation, bibliographic coupling and co-citation analysis.

Due to these reasons, bibliometric maps of this study were formed by using VOSviewer 1.6.19 software. Since there is no common agreement about which bibliometric method and tool sets are preferable in the literature, various methods and indicators were used in accordance with the purpose of this study [53,54]. In this study, performance analysis and science mapping analysis were used to provide a comprehensive review on sustainable aviation. Furthermore, co-occurrence of keywords, citation, bibliographic coupling, co-authorship, and co-citation analyses were used, respectively.

## 4 Results and discussion

### 4.1 An overview of the publication years and numbers

**Fig. 2:**
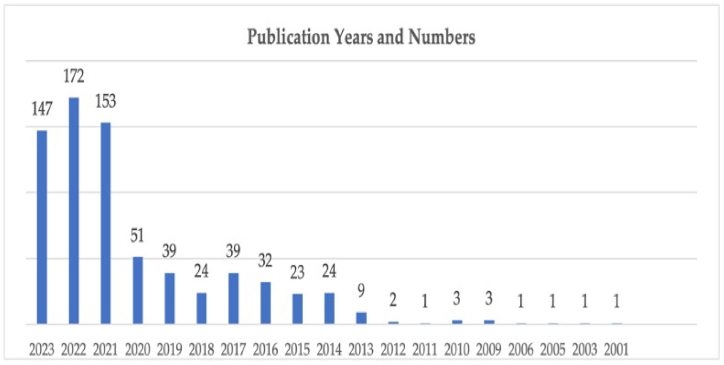
Publication years and numbers.

The bibliometric analysis of this study included 726 publications. Fig. 2 shows the number of publications published between 2001 and 2023 and indicates the development of scientific output in sustainable aviation literature. The first publication was Upham's article titled “A comparison of sustainability theory with UK and European airports policy and practice” published by Journal of Environmental Management on November 1, 2001 [55]. Considering the first study on sustainable aviation which was published in 2001, it can be asserted that sustainable aviation is a relatively new research area.

While the number of the publications was increasing considerably after 2013, there were decreases in certain years. On the other hand, the number of the publications published in the field has been increasing significantly since 2019. With regards to the number of publications, the highest number (172) was detected in 2022. Furthermore, 72 % of the publications were conducted between 2020 and 2023. 98 % of them were published between 2013 and 2023. From this point of view, it can be asserted that the importance given to the subject of sustainable aviation literature has increased in the last 10 years especially after the 2020.

### 4.2 An overview of research areas of publications

**Fig. 3:**
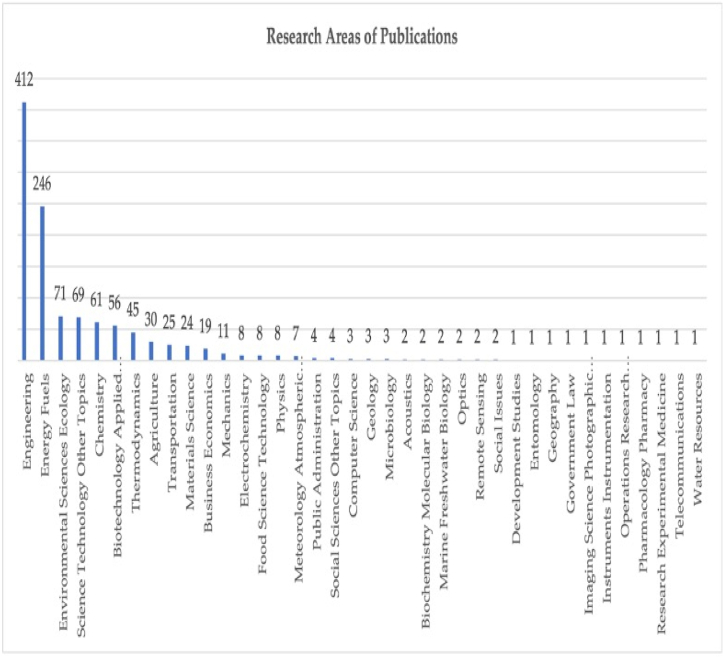
Research areas of publications.

Research areas of the publications highlights trends of sustainable aviation. Fig. 3 shows the research areas of the publications. Accordingly, 726 publications were divided into 38 research areas. “Engineering” (56.749 %) and “Energy Fuels” (33.884 %) were the main research areas in the publications on sustainable aviation, which was possibly due to the close relationship between sustainable aviation and technology.

### 4.3 An overview of publication titles

Titles of the publications were examined to identify the most productive publications in terms of high number of publications. 726 publications were published via 181 publication sources. Table 2 lists the top 10 publication sources with most publications in sustainable aviation literature. Accordingly, majority of the publications were published in International Journal of Sustainable Aviation Fuel (187 publications-25.757 %). For example, the second journal “Fuel” had 38 publications (28 publications - 5.234 %), which was followed by “Energy” (3.856 %) and “Journal of Aeronautics Astronautics and Aviation” (28 publications - 3.856 %).

**Table 2:** List of the top 10 publication sources.

No	Publication Source	Number of Publication
1.	International Journal of Sustainable Aviation	187
2.	Fuel	38
3.	Energy	28
4.	Journal of Aeronautics Astronautics and Aviation	28
5.	Energies	26
6.	Frontiers in Energy Research	24
7.	Aerospace	22
8.	Biofuels Bioproducts and Biorefining	15
9.	Aircraft Engineering and Aerospace Technology	12
10.	Journal of Cleaner Production	11

### 4.4 An overview of the top publishing researchers

**Fig. 4:**
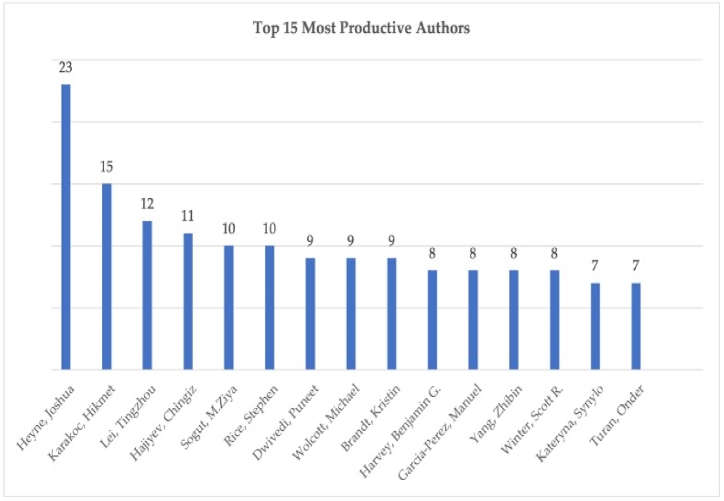
The top 15 most productive authors.

As development in a research area depends on a better comprehension of the existing limits of a scientific field, specifying the most productive authors is crucial for performance analysis [48]. Therefore, Fig. 4 shows the list of the top 15 most productive authors that make the greatest contribution to sustainable aviation literature. Based on this figure, with Heyne was the most productive author of the sustainable aviation literature thanks to his 23 publications (3.168 % of all the publications).

### 4.5 Co-occurrence of author keywords

Co-occurrence of keywords demonstrates the most common keywords generally appearing below the abstract and network links visualize keywords appearing more constantly in the same studies. Co-occurrence of keywords analyzes the main theme of the study and can ensure a perspective for future of the research area [44]. Keywords present the principal scope of the research subject and could be analyzed systematically to ensure future directions of research [56,57]. Besides, they play an important role for readability and accessibility of scientific research [58].

**Fig. 5:**
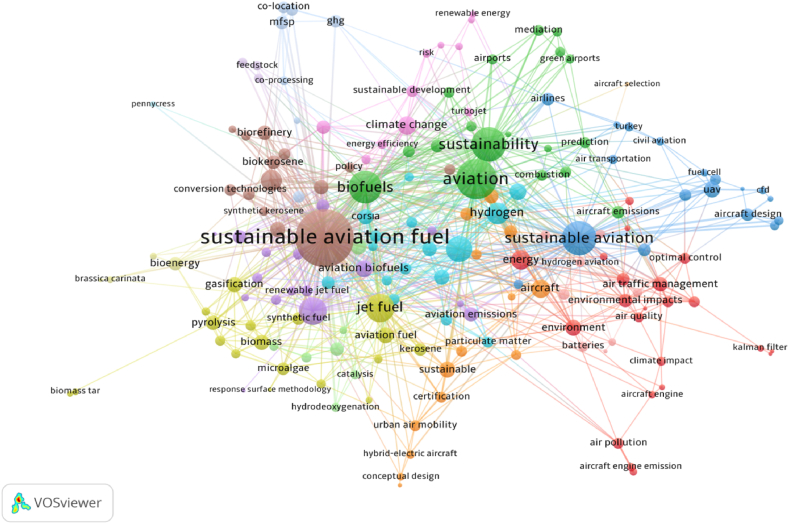
Network visualization of co-occurrence of author keywords.

This section presented the main themes in sustainable aviation research and the interaction between them from a holistic perspective. Before creating a keyword cloud, text preprocessing stages were carried out to standardize text data. Therefore, disorganizations were eliminated by gathering similar outcomes like “sustainable aviation fuel”, “sustainable aviation fuels”, “SAF”, “SAFs”, “sustainable aviation fuel (SAF)”, “sustainable aviation fuels (SAFs)”, SAF (Sustainable Aviation Fuel), SAFs (Sustainable Aviation Fuels)”, “alternative fuels”, “alternative fuel” and similarly “biofuels” and “biofuel”. In co-occurrence of author keywords analysis, minimum number of occurrences of keywords was limited to three. Of the 2423 keywords, 182 met the thresholds. Fig. 5 shows the network visualization of co-occurrence of author keywords. Table 2 lists the top 10 most frequently occurred keywords.

In network visualization, the label and size of circle indicates the item weight and “the color of an item is determined by the cluster to which the item belongs” [59]. Moreover, connections between items are represented by lines and strength of the links of the circles demonstrates by total link strength. The dimensions and lines of the clusters in Fig. 5 demonstrate the frequency of co-occurrence of author keywords in the publications. The most important circle labeled as “sustainable aviation fuels” was the most frequently occurred keyword with the highest total link strength. Furthermore, Table 3 lists the top 10 keywords in sustainable aviation literature with occurrences and total link strength numbers. As can be seen from Fig. 5 and Table 3, remarkable keywords other than “Sustainable aviation fuel” were “aviation”, “sustainability”, “sustainable aviation”, “biofuels”, “jet fuel”, “life cycle assessment”, “alternative fuels”, “hydrogen”, and “techno-economic analysis”. Similar to the literature review section, the main theme in sustainable aviation literature was sustainable aviation fuel.

**Table 3:** List of the top 10 keywords.

No	Keyword	Occurrences	Total link strength
1	Sustainable aviation fuel	168	282
2	Aviation	59	136
3	Sustainability	50	101
4	Sustainable aviation	67	98
5	Biofuels	46	91
6	Jet fuel	30	69
7	Life-cycle assessment	21	63
8	Alternative fuels	20	57
9	Hydrogen	16	39
10	Techno-economic analysis	16	38

**Fig. 6:**
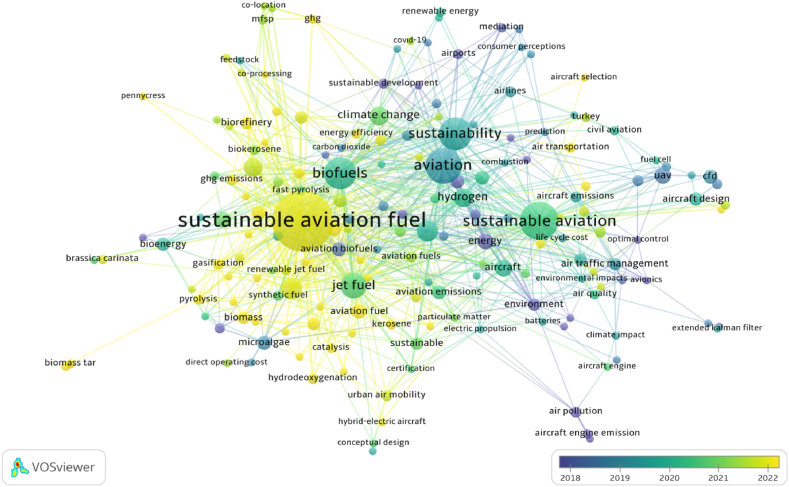
Overlay visualization of co-occurrence of author keywords.

Moreover, Fig. 6 shows the overlay visualization of co-occurrence of author keywords and developing research designs in this literature over the years. According to Pranajaya et al. [60], the overlay visualization “is a useful graphic tool for examining the temporal distribution of terms within each cluster. It employs colors ranging from purple to green to yellow to indicate the chronological occurrence of keywords”.

Considering overlay visualization of co-occurrence of author keywords, the distribution of the themes over the years has shifted from air pollution, aircraft engine emission, air quality, airports, sustainable development, and environment to sustainable aviation fuel, life-cycle analysis, hydrodeoxygenation, hybrid-electric aircraft, biorefinery, and gasification. The analysis indicated a significant change in the research focus toward newly emerging themes in a short period of time. It was determined that the current themes in the sustainable aviation literature have evolved and focused into more challenging and more technology-oriented topics. This could be associated with direct and dynamic relationship between technological developments and aviation sector. For this reason, it can be asserted that the trend themes addressed by the sustainable aviation literature would change and transform in parallel with technological developments in the future.

### 4.6 Citation analysis

#### 4.6.1 Citation analysis of authors, publications, and sources

Examining a research field necessitates an understanding of the studies contributing scientific field. Citation analysis works on the basis that authors cite publications according to the goals of their research [61]. The hypothesis here is that citations reflect intellectual connections between studies emerging when one publication quotes the other. Citation analysis is commonly used to evaluate the impact of an author, document, country or organization as it allows to quickly identify important studies in the chosen field [62,63].

**Fig. 7:**
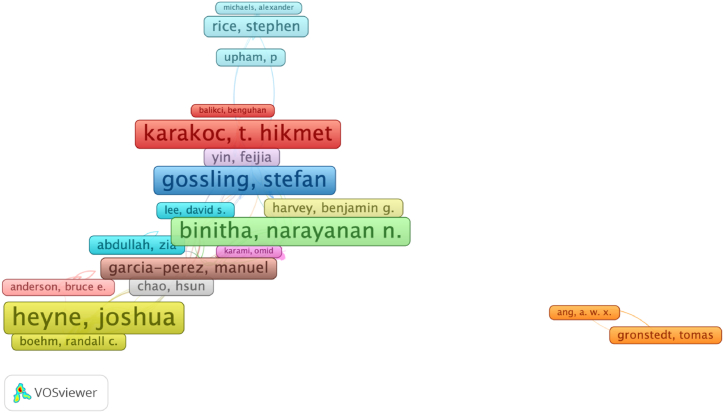
Network visualization of citation of authors.

Citation analysis of authors, publications, and sources was run to determine the most effective authors, publications, and sources in “sustainable aviation” area. To make science mapping analysis, number of publications of an author was limited to one. 1727 among 2272 authors met the criteria. Fig. 7 shows network visualization of citation analysis of authors. In terms of citation, the most productive author in sustainable aviation literature was Joshua Heyne with 306 citations. As a result of the analysis, Heyne come to the fore as both most productive author and the most cited researcher in this literature.

Table 4 shows a list of the 10 most cited publications and Table 5 shows a list of the top 10 most cited sources. Thus, documents, authors and sources inspiring most the sustainable aviation literature were emphasized.

**Table 4:** List of the top 10 most cited publications.

No	Title of Publication	Author(s)	Citation
1	Aviation biofuel from renewable resources: routes, opportunities and challenges	Hari et al. [11]	223
2	Evaluation of the potential of 9 Nannochloropsis strains for biodiesel production	Ma et al. [64]	158
3	Engineering cyanobacteria to improve photosynthetic production of alka(e)nes	Wang et al. [65]	144
4	Sustainable alternative fuels in aviation	Yilmaz and Atmanli [66]	125
5	Are technology myths stalling aviation climate policy?	Peeters et al. [67]	118
6	Hydrogen peroxide-independent production of alpha-alkenes by OleT (JE) P450 fatty acid decarboxylase	Liu et al. [68]	103
7	Bio-Based solvents for green extraction of lipids from oleaginous yeast biomass for sustainable aviation biofuel	Breil et al. [69]	86
8	Greenhouse gas emissions and land use change from jatropha curcas-based jet fuel in Brazil	Bailis and Bake [70]	82
9	Exergo-sustainability indicators of a turboprop aircraft for the phases of a flight	Aydin et al. [71]	81
10	Multi-objective optimization of aircraft flight trajectories in the ATM and avionics context	Gardi et al. [22]	71

**Table 5:** List of the top 10 most cited sources.

No	Publication Source	Publication	Citation
1	Energy	28	672
2	International Journal of Sustainable Aviation	187	454
3	Renewable & Sustainable Energy Reviews	10	380
4	Fuel	38	331
5	Bioresource Technology	6	292
6	Transportation Research Part D-Transport and Environment	7	185
7	Global Change Biology Bioenergy	10	178
8	Biomass & Bioenergy	8	165
9	Energies	26	121
10	Aerospace	22	120

The top 10 most cited studies focused mostly on sustainable aviation fuel, its production and its use in aviation. Seven of these studies were related to these subjects [11,[64], [65], [66],[68], [69], [70]]. In terms of publication year, it was found that only one study was published in 2010, and the other nine studies were published in 2013 and the following years.

Accordingly, the most cited study with 223 citations reviewed was the aviation biofuel from renewable resources by Hari et al. [11]. When the studies were evaluated according to the number of citations, Hari et al.’s [11] and Yilmaz and Atmanli's [66] study focused on general issues on sustainable aviation fuels, other publications mostly examined more specific issues [22,64,65,[68], [69], [70], [71]]. For example, in their study, Ma et al. [64] evaluated “Potential of 9 Nannochloropsis strains for biodiesel production” while Wang et al.’s [65]study examined “Engineering cyanobacteria to improve photosynthetic production of alka(e)nes”.

Table 5 lists the top ten most cited publication sources. In terms of the most cited sources, “Energy” is the top one with 672 citations, but its publication number is relatively low. On the other hand, contrary to its overwhelming number, “International Journal of Sustainable Aviation” was the second source with 454 citations. Moreover, with regards to publications’ titles and sources, it can be asserted that literature of sustainable aviation mainly benefits from environment, energy, renewable and sustainable energy and bio-technology oriented sources.

#### 4.6.2 Most cited countries and organizations

**Fig. 8:**
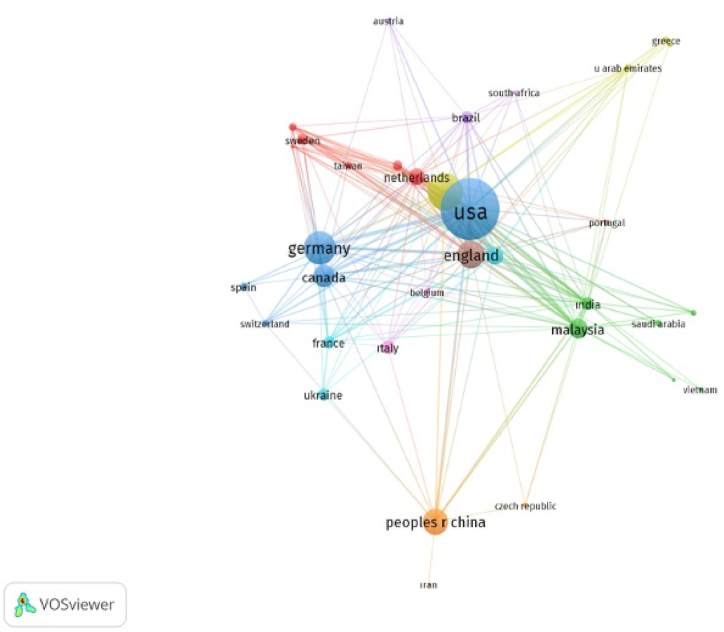
Network visualization of citation analysis of countries.

To figure out demographic distribution of the research on sustainable aviation and to find out the most cited countries and organizations, both performance and science mapping analyses were used. To carry out science mapping analysis, citation of countries and organizations was limited to minimum three publications and 20 citations. 36 out of a total of 75 countries have minimum three publications and 20 citations. Moreover, 86 out of a total of 812 organizations met the thresholds. Citation analysis of countries and organizations was demonstrated via network visualization. According to Fig. 8, the most cited country was the U.S.A. In terms of links and clusters among countries, the researchers from the U.S.A. frequently conducted joint publications with Germany, Canada, Spain, and Switzerland.

Moreover, Table 6 lists the top cited countries and gives information about their publications and citation numbers. In terms of the citations, the U.S.A. (1590 citations), Peoples R China (983 citations) and England (730 citations) were the top three in the list. On the other hand, in terms of number of publications, the majority of publications were conducted in the U.S.A. (187 publications), which was followed by Türkiye (92 publications) and Germany (79 publications).

**Table 6:** List of the top 10 most cited countries.

No	Country	Publication	Citation
1	USA	187	1590
2	Peoples R China	57	983
3	England	62	730
4	Germany	79	627
5	Australia	31	622
6	Türkiye	92	612
7	Canada	45	441
8	Malaysia	38	398
9	Netherlands	30	367
10	India	20	348

Note: Some publications and citations may be given by authors belonging to more than one country because such authors studying together in the same publication are from different nations.

**Fig. 9:**
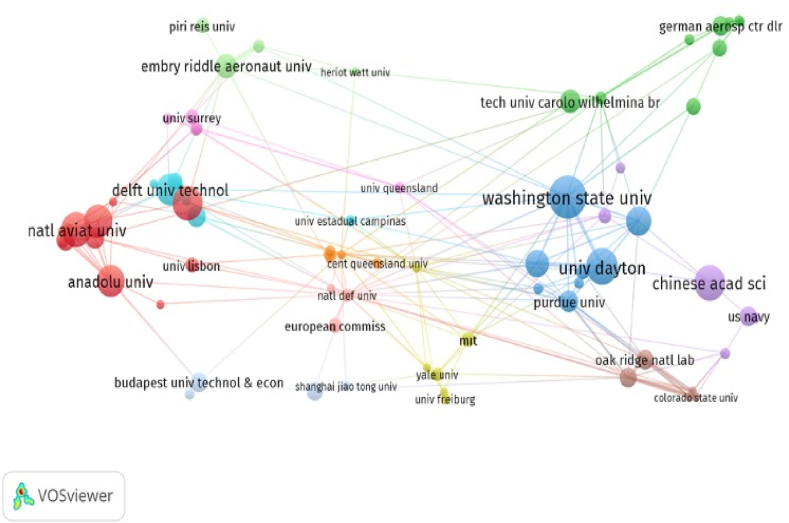
Network visualization of citation analysis of organizations.

In terms of citation analysis of organizations, the size of the circles demonstrates the citation analysis of 86 organizations (out of 812) in Fig. 9 Furthermore, the number of publications and citations of organizations are present in Table 7. In terms of the number of publications, Washington State University was the most contributing organization to literature on sustainable aviation (26 publications), which was followed by University of Dayton (21 publications) and Chinese Academy of Sciences (20 publications). On the other hand, in terms of citation numbers, Chinese Academy of Sciences was the most cited organization with 245 citations. The results of the citation analysis of countries and organizations indicated that the USA emerged as the most influential country in terms of publication and citation number while Chinese Academy of Sciences was most influential organization in terms of citation number.

**Table 7:** List of the top 10 organizations contributing to literature on sustainable aviation.

No	Organization	Publication	Citation
1	Washington State University	26	157
2	University of Dayton	21	245
3	Chinese Academy of Sciences	20	716
4	Universiti Putra Malaysia	19	94
5	National Aviation University	19	54
6	Eskisehir Technical University	18	51
7	Anadolu University	17	198
8	Delft University of Technology	17	145
9	National Renewable Energy Laboratory	15	93
10	Pacific Northwest National Laboratory	14	79

### 4.7 Bibliographic coupling analysis

#### 4.7.1 Bibliographic coupling analysis of authors

Bibliographic coupling occurs when two publications cite the same third publication. It works on the assumption that the contents of two publications sharing common references are also similar [72]. Since the degree of centrality of an author is measured by the number of connections within a research network, authors with the strongest citation relationships are more prominent in the citation network and participate more in discussions [44]. As the number of common references increases, the strength of bibliographic links also increases.

In the bibliographic coupling analysis of authors, a minimum of two publications per author were accepted as a criterion. Of the 2272 authors, 426 met the thresholds. Accordingly, Table 8 shows the top 10 authors with the highest connection strengths based on the total link strength of the bibliographic coupling analysis. According to the result of the analysis, Heyne, who was ranked as the first among the most productive and most cited authors, also became the first author (1023 links) in terms of total link strength of the bibliographic analysis. His studies were published between 2019 and 2023 [73]. Heyne, studying on energy and fuels, engineering, chemistry, thermodynamics and physics [74], examined mostly on production of sustainable aviation fuel and its use in aviation [[75], [76], [77], [78], [79], [80], [81], [82], [83], [84], [85], [86]].

**Table 8:** List of the top 10 authors in bibliographic coupling link strength.

No	Author	Citation	Total Link Strength
1	Heyne, Joshua	306	1023
2	Wolcott, Mitchael	91	740
3	Garcia-Perez, Manuel	84	725
4	Brandt, Kristin	76	679
5	Dwivedi, Puneet	54	610
6	Afonso, Frederico	22	595
7	Ferreira, Ana	22	595
8	Lau, Fernando	22	595
9	Ribeiro, Ines	22	595
10	Suleman, Afzal	22	595

### 4.8 Co-authorship analysis

#### 4.8.1 Co-authorship analysis of organizations

Co-authorship analysis investigates the interactions between scholars in a study area [48]. “Co-authorship, a proxy of research collaboration, is a key mechanism that links different sets of talent to produce a research output” [87]. Fig. 9 shows collaborative network among organizations. To carry out science mapping analysis, minimum number of publications of an organization was limited to three publications and [20] citations [86]. out of a total 812 organizations met the thresholds [50]. of [86] organizations were determined to connect to each other and [36] organizations were not connected. Therefore [36], organizations were excluded from the analysis. Co-authorship analysis of organizations was demonstrated via network visualization. The analysis revealed the presence of eight clusters representing [50] connected organizations.

**Fig. 10:**
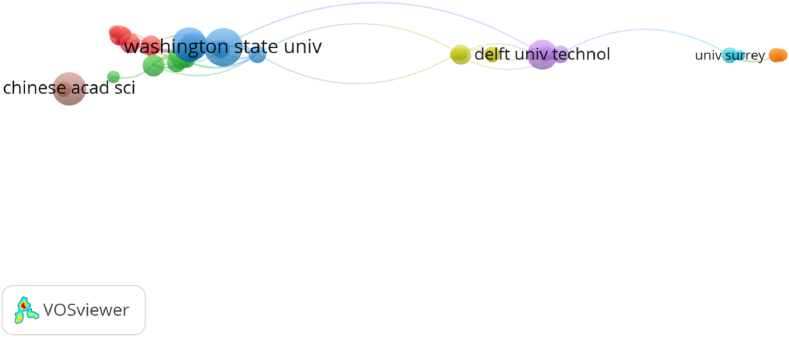
Network visualization of co-authorship analysis of organizations.

Similar to the results of citation analysis of countries and organizations, Washington State University had the highest connections with other organizations. Furthermore, as seen in Fig. 10, co-authorship networks of organizations were relatively weak. Therefore, in the dynamic global conjuncture where technological developments have gained a new dimension day by day, more interactive co-authorship collaboration including more organizations may be needed to develop sustainable aviation literature as more knowledge and technology oriented in a multidimensional manner from different perspectives.

### 4.9 Co-citation analysis

#### 4.9.1 Co-citation analysis of cited references

Unlike citation analysis, co-citation analysis demonstrates the studies citing a particular pair of references by gathering data from databases and benefitting from analytical maps and graphical presentation techniques that could be utilized to generate empirical maps of leading authors in various fields of science [88]. Similarity of content can be reflected through co-citation analysis [89]. Thus, subject, author groups and their connection can be determined. “A co-citation link is a link between two items that are both cited by the same document” [59]. The intellectual structure of the research area can be understood via co-citation analysis. Moreover, the use of co-citation analysis to figure out the current state of the literature may be beneficial in determining its future areas.

**Fig. 11:**
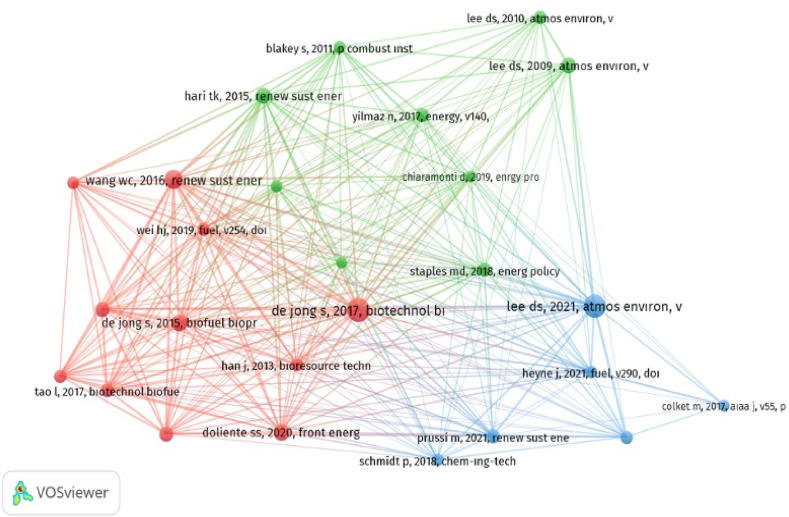
Network visualization of co-citation analysis of the cited references.

Science mapping of co-citation analysis was used on sustainable aviation literature to determine similar topic fields and examine scientific feedback of other scholars. In the analysis, minimum number of citations of a cited reference was selected as 20. Of the 30912 cited references, 26 met the thresholds. The analysis demonstrated three research clusters (Fig. 11). The size of the nodes shows the size of the co-citations of the references. The lines between the circles show co-citation relationships. The thickness of the lines indicates that the references are in strong cooperation regarding co-citation. Clusters with the same color signify that the references are related to each other. Moreover, Table 9 shows the fundamental subjects and related references of these clusters.

**Table 9:** Fundamental subjects and related references of the research clusters.

Cluster	Broad Theme	References
Cluster 1 A total of 11 publications	Economic, environmental, technological and techno-economic assessments on sustainable aviation fuel and its production, life-cycle assessment, and supply chain components of bio-aviation fuel	de Jong et al. [90], de Jong et al. [91], Doliente et al. [92], Geleynse et al. [93], Gutiérrez-Antonio et al. [94] Han et al. [95], Mawhood et al. [96], Pearlson et al. [97], Tao et al. [98], Wang and Tao [99], Wei et al. [100]
Cluster 2 A total of 9 publications	Evaluation of the effects of aviation sectors on climate change, assessment on sustainable alternative fuels, sustainable aviation fuel effects on emission mitigation, biofuels from renewable sources	Blakey et al. [101], Chiaramonti [102], Hari et al. [11], Kousoulidou and Lonza [103], Lee et al. [104], Lee et al. [105], Staples et al. [106], Wang et al. [107] Yilmaz and Atmanli [66]
Cluster 3 A total of 6 publications	Jet fuels combustion program, sustainable aviation fuel procedures, effects of aviation on anthropogenic climate change, CORSIA, renewable fuel options, effects of cleaner burning aviation fuels on contrail cloudiness	Colket et al. [108], Heyne et al. [78], Lee et al. [109], Prussi et al. [110], Schmidt et al. [111], Voigt et al. [112]

According to Table 9, Cluster 1 consisted of 11 publications and the examined issues in the cluster were mostly on economic, environmental, technological and techno-economic assessments on sustainable aviation fuel and its production. Accordingly, de Jong et al. [90], made a techno-economic comparison about “the feasibility of short-term production strategies for renewable jet fuels”. Doliente et al. [92] assessed supply chain components of bio-aviation fuel. Geleynse et al. [93], Pearlson et al. [97], and Tao et al. [98] made techno-economic assessments on “the alcohol-to-jet conversion pathway for drop-in biofuels”, “hydroprocessed renewable esters and fatty acids for production of jet fuel”, and “hydroprocessed renewable jet fuel” respectively. Gutiérrez-Antonio et al. [94], Mawhood et al. [96] and Wei et al. [100] studied on the production processes and pathways of renewable jet fuel. In their studies, de Jong et al. [91] and Han et al. [95] made an evaluation on life-cycle analysis of sustainable aviation fuels. While de Jong et al. [91] studied on “life-cycle analysis of greenhouse gas emissions from renewable jet fuel production”, Han et al. [95]focused on “life-cycle analysis of bio-based aviation fuels. Wang and Tao [99] studied on “Bio-jet fuel conversion technologies”.

Cluster 2 consisted of 9 publications and the addressed topics were related to the evaluation of the effects of aviation sector on climate change and assessment on sustainable alternative fuels. Lee et al. [104,105] addressed the aviation's effects on climate change issue from different dimensions via their studies titled “aviation and global climate change in the 21st century” and “transport impacts on atmosphere and climate: aviation”, respectively. Furthermore, the other main broad topic in Cluster 2 was determined as an assessment on sustainable alternative fuels. Accordingly, while Blakey et al. [101] made an assessment about “aviation gas turbine alternative fuels”, Chiaramonti [102], Kousoulidou and Lonza [103], and Wang et al. [107] studied on sustainable aviation fuels, biofuels in aviation and biomass-derived aviation fuels, respectively. Similarly, Yilmaz and Atmanli [66] studied “sustainable alternative fuels in aviation”. The third topic which was mainly addressed in Cluster 2 was specified as effects of sustainable aviation fuel on emission mitigation. Moreover, Staples et al. [106] examined “aviation CO_2_ emission reductions from the use of alternative jet fuels”. In addition, the last topic in Cluster 2 was determined as biofuels from renewable sources based on Hari et al.’s [11] studied on “aviation biofuel from renewable resources: routes, opportunities and challenges”.

Cluster 3 covered 6 publications emphasizing principally on jet fuels combustion program, sustainable aviation procedures, effects of aviation on anthropogenic climate change, CORSIA and renewable and cleaner fuel options topics. Accordingly, in their study, Colket et al. [108] conducted “an overview of the National Jet Fuels Combustion Program led by the Federal Aviation Administration, the U.S. Air Force Research Laboratory, and the NASA”. Heyne et al. [78] gave information about “Sustainable aviation fuel prescreening tools and procedures” and recommended two-tiered prescreening process. Lee et al. [109] examined the global aviation's impacts on climate change via their studies on “The contribution of global aviation to anthropogenic climate forcing for 2000 to 2018”. Prussi et al. [110] assessed CORSIA founded by International Civil Aviation Organization (ICAO) aimed to mitigate aviation greenhouse gas emissions. Schmidt et al. [111] and Voigt et al. [112] studied on renewable fuel options for aviation and cleaner burning aviation fuels effects on contrail cloudiness respectively in their studies. As in the outputs of literature analysis and other analysis studies conducted on sustainable aviation, co-citation analysis of cited references and fundamental subjects and related references of the research clusters predominantly focused on the examination of sustainable aviation fuel, its production and its use in aviation.

## 5 Conclusions

### 5.1 Key findings

This study revealed the following key results: (1) The number of studies published on sustainable aviation has been increasing significantly since 2020. This can be associated with the increasing importance attached to sustainability in almost every field in the world and the contributions of dynamic technological developments on scientific field. (2) Issues on sustainable aviation are basically on examination of sustainable aviation fuel, its production and its use in aviation. (3) The research on sustainable aviation were mainly conducted by the authors from the universities in the U.S.A. (4) The themes addressed by the studies on sustainable aviation are gradually evolving into more complicated and complex issues and technology-oriented areas.

### 5.2 Implications for theory and practices

The quantitative bibliometric analysis method was applied in this study to examine the retrospective features of research on sustainable aviation. This study is presumably one of the first and original studies to contextualize a bibliometric analysis of sustainable aviation literature. Therefore, identifying key areas and findings in the field of sustainable aviation through bibliometric analysis can offer researchers a clearer picture of studies on subject and may guide them on how to gain insight for future studies. In addition, this study provides practical implications as well as academic implications. Airlines, governments, oversight agencies, and business enterprises can benefit from its findings to make decisions. The findings of the study may contribute to raise awareness about sustainable aviation and may help to make more knowledge-based decisions and future projections on sustainable aviation issues.

### 5.3 Limitations and future research directions

As is the case in other studies, this study has some limitations. Firstly, in the scope of the study, publications other than English and indexes except for SCI, SSCI and ESCI were excluded. Secondly, Web of Science database was used to search the literature and to make an analysis, and VOSviewer was used as software. Publications from other databases like Scopus, Google Scholar, Microsoft Academic and Dimensions were not included. Thirdly, the sample size is limited to a single search key. Therefore, future studies are recommended to investigate this subject from different databases, indexes, software, and analyses and including a larger sample size and more keywords to contribute to the literature.

Moreover, statistics and machine learning methods have become more integrated into workflows thanks to advances in digital technology. “Airlines have some of the most sophisticated technology ever created and are becoming even more technology oriented, now encompassing a wide range of interconnected industry ecosystems” [113]. On the other hand, digital technology has still been developing and “research efforts are needed to both overcome fundamental machine learning problems and apply novel methods in the aviation field” [114]. Therefore, as the impact and importance of digital technology on the aviation industry increases, the future studies on aviation and sustainable aviation shall focus on more specific and complicated topics and methods especially on the relationship between sustainability, aviation, and digital technology. Therefore, in the light of current information and technological and innovative progresses, it may be benefical to establish more co-authorship relationships to develop sustainable aviation literature with more in-depth examinations and different dimensions.

## Data availability statement

Question.

Response.

Data Availability.

Sharing research data helps other researchers evaluate your findings, build on your work and to increase trust in your article. We encourage all our authors to make as much of their data publicly available as reasonably possible. Please note that your response to the following questions regarding the public data availability and the reasons for potentially not making data available will be available alongside your article upon publication.

Has data associated with your study been deposited into a publicly available repository?

No.

Please select why. Please note that this statement will be available alongside your article upon publication. As follow-up to “Data Availability”. Sharing research data helps other researchers evaluate your findings, build on your work and to increase trust in your article. We encourage all our authors to make as much to their data publicly available as reasonably possible. Please note that your response to the following questions regarding the public data availability and the reasons for potentially not making data available will be available alongside your article upon publication.

Data will be made available on request.

## CRediT authorship contribution statement

**Fatma Cande Yaşar Dinçer:** Writing – review & editing, Writing – original draft, Formal analysis. **Gözde Yirmibeşoğlu:** Writing – review & editing, Writing – original draft, Supervision, Conceptualization. **Yasemin Bilişli:** Writing – original draft, Methodology, Formal analysis, Conceptualization. **Emel Arık:** Methodology, Investigation, Formal analysis, Conceptualization. **Hakkı Akgün:** Methodology, Investigation, Formal analysis, Conceptualization.

## Declaration of competing interest

The authors declare that they have no known competing financial interests or personal relationships that could have appeared to influence the work reported in this paper.

## References

[1] UNFCC (2017).

[2] Milner M.M., Bush D.Z., Anania E.C., Ito T., Marte D.A., Rice S., Winter S.R. (2019). Cultural and political attitudes towards paying to support airport sustainability projects. Int. J. Sustain. Aviation.

[3] Merdivenci F., Erturgut R., Coşkun A.E. (2021). A software development application for sustainable airport performance analysis. LogForum.

[4] Yaşar Dinçer F.C., Yirmibeşoğlu G. (2024). Constraints on women pilots in airline industry: a rising sector of international trade. J. Air Transport. Manag..

[5] Turkish Airlines (2022). Sustainable avaition. https://terminal.turkishairlines.com/en/sustainable-aviation/.

[6] Janic M. (2002). Methodology for assessing sustainability of an air transport system. J. Air Transport..

[7] Seyam S., Dincer I., Agelin-Chaab M. (2021). Investigation of two hybrid aircraft propulsion and powering systems using alternative fuels. Energy.

[8] Murphy H.T., O'Connell D.A., Raison R.J., Warden A.J., Booth T.H., Herr A., Braid A.L., Crawford D.F., Hayward J.A., Jovanovic T., McIvor J.G., O'Connor M.H., Poole M.L., Prestwidge D., Raisbeck-Brown N., Lye L. (2015). Biomass production for sustainable aviation fuels: a regional case study in Queensland. Renew. Sustain. Energy Rev..

[9] Peters M.A., Alves C.T., Onwudili J.A. (2023). A review of current and emerging production technologies for biomass-derived sustainable aviation fuels. Energies.

[10] Okolie J., Awotoye D., Tabat M.E., Ogbaga C.C., Güleç F., Oboirien B. (2023). Multi-criteria decision analysis for the evaluation and screening of sustainable aviation fuel production pathways. iScience.

[11] Hari T.K., Yaakob Z., Binitha N.N. (2015). Aviation biofuel from renewable resources: routes, opportunities and challenges. Renew. Sustain. Energy Rev..

[12] Cruz G., Silva A.V.S., Da Silva J.B.S., Caldeiras R.D., de Souza M.E.P. (2020). Biofuels from oilseed fruits using different thermochemical processes: opportunities and challenges. Biofuels Bioproducts & Biorefining-Biofpr.

[13] Yusaf T., Fernandes L., Abu Talib A.R., Altarazi Y.S.M., Alrefae W., Kadirgama K., Ramasamy D., Jayasuriya A., Brown G., Mamat R., Dhahat H.A., Benedict F., Laimon M. (2022). Sustainable aviation-hydrogen is the future. Sustainability.

[14] Dincer I., Acar C. (2016). A review on potential use of hydrogen in aviation applications. Int. J. Sustain. Aviation.

[15] Degirmenci H., Uludağ A., Ekici S., Karakoc T.H. (2023). Challenges, prospects and potential future orientation of hydrogen aviation and the airport hydrogen supply network: a state-of-art review. Prog. Aero. Sci..

[16] Undavalli V., Olatunte O.B.G., Boylu R., Wei C., Haeker L., Hamilton J., Khandelwal B. (2022).

[17] Hasan M.A., Mamun A.A., Rahman S.M., Malik K., Al Amran M.I.U., Khondaker A.N., Reshi O., Tiwari S.P., Alismail F.S. (2021). Climate change mitigation pathways for the aviation sector. Sustainability.

[18] Cabrera E., de Sousa J.M.M. (2022). Use of sustainable fuels in aviation—a review. Energies.

[19] Brooker P. (2006). Civil aircraft design priorities: air quality? climate change? noise?. Aeronaut. J..

[20] Wang J.Z. (2014). High-order computational fluid dynamics tools for aircraft design. Phil. Trans. Math. Phys. Eng. Sci..

[21] Abu Salem K., Palaia G., Quarta A.A. (2023). Review of hybrid-electric aircraft technologies and designs: critical analysis and novel solutions. Prog. Aero. Sci..

[22] Gardi A., Sabatini R., Ramasamy S. (2016). Multi-objective optimization of aircraft flight trajectories in the ATM and avionics context. Prog. Aero. Sci..

[23] Ranasinghe K., Guan K., Gardi A., Sabatini R. (2019). Review of advanced low-emission technologies for sustainable aviation. Energy.

[24] Bravo-Mosquera P.D., Catalano F.M., Zingg D.W. (2022). Unconventional aircraft for civil aviation: a review concepts and design. Prog. Aero. Sci..

[25] Zhang J., Roumeliotis I., Zolotas A. (2022). Sustainable aviation electrification: a comprehensive review of electric propulsion system architectures, Energy Management, and Control. Sustainability.

[26] Keiser D., Schnoor L.H., Pupkes B., Freitag M. (2023). Life cycle assessment in aviation: a systematic literature review of applications, methodological approaches and challenges. J. Air Tansport Manage..

[27] Hu Y.J., Yang L., Cui H., Wang H., Li C., Tang B.J. (2022). Strategies to mitigate carbon emissions for sustainable aviation: a critical review from a life-cycle perspective. Sustain. Prod. Consum..

[28] Ritchie H. (2020). Climate change and flying: what share of global CO2 emissions come from aviation?. https://ourworldindata.org/co2-emissions-from-aviation.

[29] IATA (2023). https://www.iata.org/en/services/environmental-solutions/.

[30] (2021). IATA, Net-zero carbon emissions by 2050. https://www.iata.org/en/pressroom/pressroom-archive/2021-releases/2021-10-04-03/.

[31] Walker S., Cook M. (2009). The contested concept of sustainable aviation. Sustain. Dev..

[32] IATA (2024). What is SAF?. https://www.iata.org/contentassets/d13875e9ed784f75bac90f000760e998/saf-what-is-saf.pdf.

[33] Boeing (April 2023). https://sustainabilitytogether.aero/wp-content/uploads/2023/05/Hydrogen_FactSheetR4_051523.pdf.

[34] Finnveden G., Hauschild M.Z., Ekvall T., Guinée J., Heijungs R., Hellweg S., Koehler A., Pennington D., Suh S. (2009). Recent developments in life cycle assessment. J. Environ. Manag..

[35] van Eck N.J., Waltman L. (2010). Software survey: VOSviewer, a computer program for bibliometric mapping. Scientometrics.

[36] Page M.J., McKenzie J.E., Bossuyt P.M., Boutron I., Hoffmann T.C., Mulrow C.D., Shamseer L., Tetzlaff J.M., Akl E.A., Brennan S.E., Chou R., Glanville J., Grimshaw J.M., Hrobjartsson A., Lalu M.M., Li T., Loder E.W., Mayo-Wilson E., McDonald S., McGuinness L.A., Stewart L.A., Thomas J., Tricco A.C., Welch V.A., Whiting P., Moher D. (2021). The PRISMA 2020 statement: an updated guideline for reporting systematic reviews. BMJ.

[37] PRISMA (2024). http://prisma-statement.org/prismastatement/flowdiagram.aspx.

[38] Dixit A., Jakhar S.K. (2021). Airport capacity management: a review and bibliometric analysis. J. Air Transport. Manag..

[39] Merigó J.M., Gil-Lafuente A.M., Yager R.R. (2015). An overview of fuzzy research with bibliometric indicators. Appl. Soft Comput. J..

[40] Merigó J.M., Yang J.B. (2017). A bibliometric analysis of operations research and management science. Omega.

[41] Bravo A., Vieira D., Rebello T.A. (2022). The origins, evolution, current state, and future of green products and consumer research: a bibliometric analysis. Sustainability.

[42] Cobo M.J., López-Herrera A.G., Herrera-Viedma E., Herrera F. (2021). An approach for detecting, quantifying, and visualizing the evolution of a research field: a practical application to the Fuzzy Sets Theory field. Journal of Informetrics.

[43] Noyons E.C.M., Moed H.F., Van Raan A.F.J. (1999). Integrating research performance analysis and science mapping. Scientometrics.

[44] Donthu N., Kumar S., Mukherjee D., Pandey N., Lim W.M. (2021). How to conduct a bibliometric analysis: an overview and guidelines. J. Bus. Res..

[45] Small H. (1999). Visualizing science by citation mapping. J. Am. Soc. Inf. Sci..

[46] De la Torre Bayo J.J., Martín Pascual J., Torres Rojo J.C., Zamorano Toro M. (2022). Waste to energy from municipal wastewater treatment plants: a science mapping. Sustainability.

[47] Gupta N., Chakravarty R. (2021). Trens in IoT research: a bibliometric and science mapping analysis of internet of things. Libr. Philos. Pract..

[48] Bakır M., Özdemir E., Akan Ş., Atalık Ö. (2022). A bibliometric analysis of airport service quality. J. Air Transport. Manag..

[49] Mulet-Forteza C., Genovart-Balaguer J., Mauleon-Mendez E., Merigó J.M. (2018). A bibliometric research in the tourism, leisure and hospitality fields. J. Bus. Res..

[50] Abrizah A., Zainab A.N., Kiran K., Raj R.G. (2012). LIS journals scientific impact and subject categorization: a comparison between Web of Science and Scopus. Scientometrics.

[51] Yu Y., Li Y., Zhang Z., Gu Z., Zhong H., Zha Q., Yang L., Zhu C., Chen E. (2020). A bibliometric analysis using VOSviewer of publications on COVID-19. Ann. Transl. Med..

[52] Moral-Muñoz J.A., Herrera-Viedma E., Santisteban-Espejo A., Cobo M.J. (2020). Software tools for conducting bibliometric analysis in science: an up-to-date review. El profesional de la informa- coin.

[53] Cancino C., Merigo J.M., Coronado F., Dessouky Y., Dessouky M. (2017). Forty years of computers & industrial engineering: a bibliometric analysis. Comput. Ind. Eng..

[54] Tanrıverdi G., Bakır M., Merkert R. (2020). What can we learn from the JATM literature for the future of aviation post Covid-19? a bibliometric and visualization analysis. J. Air Transport. Manag..

[55] Upham P. (2001). A comparison of sustainability theory with UK and European airports policy and practice. J. Environ. Manag..

[56] Su H.N., Lee P.C. (2020). Mapping knowledge structure by keyword co-occurrence: a first look at journal papers in Technology Foresight. Scientometrics.

[57] Cheng F.F., Huang Y.W., Yu H.C., Wu C.S. (2018). Mapping knowledge structure by keyword co-occurrence and social network analysis: evidence from Library Hi Tech between 2006 and 2017. Libr. Hi Technol..

[58] Gastel B., Day R.A. (2012).

[59] van Eck N.J., Walltman L. (2023). https://www.vosviewer.com/documentation/Manual_VOSviewer_1.6.19.pdf.

[60] Pranajaya E., Alexandri M.B., Chan A., Hermanto B. (2024). Examining the influence of financial inclusion on investment decision: a bibliometric review. Heliyon.

[61] Danvila-del-Valle I., Estévez-Mendoza C., Lara F.J. (2017). Human resources training: a bibliometric analysis. J. Bus. Res..

[62] Suban S.A. (2023). Bibliometric analysis on wellness tourism – citation and co-citation analysis. Int. Hosp. Rev..

[63] Krabokoukis T., Polyzos S. (2023). A bibliometric analysis of integrating tourism development into urban planning. Sustainability.

[64] Ma Y., Wang Z., Yu C., Yin Y., Zhou G. (2014). Evaluation of the potential of 9 Nannochloropsis strains for biodiesel production. Bioresour. Technol..

[65] Wang W.H., Liu X., Lu X. (2013). Engineering cyanobacteria to improve photosynthetic production of alka(e)nes. Biotechnol. Biofuels.

[66] Yilmaz N., Atmanli A. (2017). Sustainable alternative fuels in aviation. Energy.

[67] Peeters P., Higham J., Kutzner D., Cohen S., Gössling S. (2016). Are technology myths stalling aviation climate policy?. Transport. Res. Transport Environ..

[68] Liu Y., Wang C., Yan J., Zhang W., Guan W., Lu X., Li S. (2014). Hydrogen peroxide-independent production of alpha-alkenes by OleT(JE) P450 fatty acid decarboxylase. Biotechnol. Biofuels.

[69] Breil C., Meullemiestre A., Vian M., Chemat F. (2016). Bio-Based solvents for green extraction of lipids from oleaginous yeast biomass for sustainable aviation biofuel. Molecules.

[70] Bailis R.E., Bake J.E. (2010). Greenhouse gas emissions and land use change from jatropha curcas-based jet fuel in Brazil. Environ. Sci. Technol..

[71] Aydin H., Turan O., Karakoc T.H., Midilli A. (2013). Exergo-sustainability indicators of a turboprop aircraft for the phases of a flight. Energy.

[72] Kessler M.M. (1963). Bibliographic coupling between scientific papers. Am. Doc..

[73] (2024). Web of Science.

[74] (2024). Web of Science.

[75] Peiffer E., Heyne J.S., Colket M.B. (2019). Sustainable aviation fuels approval streamlining: auxiliary power unit lean blowout testing. AIAA J..

[76] Kosir S., Stachler R., Heyne J., Hauck F. (2020). High performance jet fuel optimization and uncertainty analysis. Fuel.

[77] Huq N.A., Hafenstine G.R., Huo X.C., Nyugen H., Tifft S.M., Conklin D.R., Stück D., Stunkel J., Yang Z.B., Heyne J.S., Wiatrowski M.R., Zhang Y.M., Tao L., Zhu J.Q., McEnally C.S., Christensen E.D., Hays C., Vall Alsburg K.M., Unocic K.A., Meyer H.M., Albullah Z., Vardon D.R. (2021). Toward net-zero sustainable aviation fuel with waste-derived volatile fatty acids. Proc. Natl. Acad. Sci. U.S.A..

[78] Heyne J., Rauch B., Le Clerq P., Colket M. (2021). M. Sustainable aviation fuel prescreening tools and procedures. Fuel.

[79] Kramer S., Andac G., Heyne J., Ellsworth J., Herzig P., Lewis K.C. (2022). Perspectives on fully synthesized sustainable aviation fuels: direction and opportunities. Front. Energy Res..

[80] Cronin D.J., Subramaniam S., Brady C., Cooper A., Yang Z.B., Heyne J., Drennan C., Ramasamy K.K., Thorson M.R. (2022). Sustainable aviation fuel from hysrothermal liquefaction of wet wastes. Energies.

[81] Faulhaber C., Borland C., Boehm R., Heyne J. (2023). Measurements of nitrile rubber absorption of hydracarbons: trenns for sustainable aviation compatilibity. Energy Fuel..

[82] Ruan H., Qin Y.L., Heyne J., Gieleciak R., Feng M.Q., Yang B. (2019). Chemical compositions and properties of lignin-based jet fuel range hydracarbons. Fuel.

[83] Stachler R., Heyne J., Stouffer S., Miller J. (2020). Lean blowoff in toroidal jet-stirred reactor: implications for alternative fuel approval and potential mechanisms for autoignition and extinction. Energy Fuel..

[84] Kosir S., Heyne J., Graham J. (2020). A machine learning for drop-in volume swell characteristics of sustainable aviation fuel. Fuel.

[85] Boehm R.C., Scholla L.C., Heyne J.S. (2021). Sustainable alternative fuel effects on energy consumption of jet engines. Fuel.

[86] Vardon D.R., Sherbacow B.J., Guan K.Y., Heyne J.S., Abdullah Z. (2022). Realizing “net-zero-carbon” sustainable aviation fuel. Jolue.

[87] Kumar S. (2015). Co-authorship networks: a review of the literature. Aslib J. Inf. Manag..

[88] McCain K.W. (1990). Mapping authors in intellectual space: a technical overview. J. Am. Soc. Inf. Sci..

[89] Chou C.H., Ngo S.L., Tran P.P. (2023). Renewable energy integration for sustainable economic growth: insights and challenges via bibliometric analysis. Sustainability.

[90] de Jong S., Hoefnagels R., Faaij A., Slade R., Mawhood R., Junginger M. (2015). The feasibility of short-term production strategies for renewable jet fuels – a comprehensive techno-economic comparison. Biofuels, Bioproducts and Biorefining.

[91] de Jong S., Antonissen K., Hoefnagels R., Lonza L., Wang M., Faaij A., Junginger M. (2017). Life-cycle analysis of greenhouse gas emissions from renewable jet fuel production. Biotechnol. Biofuels.

[92] Doliente S.S., Narayan A., Tapia J.F.D., Samsatli N.J., Zhao Y., Samsatli S. (2020). Bio-aviation fuel: a comprehensive review and analysis of the supply chain components. Front. Energy Res..

[93] Geleynse S., Brandt K., Wolcott M., Garcia-Perez M., Zhang X. (2018). The alcohol-to-jet conversion pathway for drop-in biofuels: techno-economic evaluation. ChemSusChem.

[94] Gutiérrez-Antonio C., Gómez-Castro F.I., de Lira-Flores J.A., Hernández S. (2017). A review on the production processes of renewable jet fuel. Renew. Sustain. Energy Rev..

[95] Han J., Elgowainy A., Cai H., Wang M.Q. (2013). Life-cycle analysis of bio-based aviation fuels. Bioresour. Technol..

[96] Mawhood R., Gazis E., de Jong S., Hoefnagels R., Slade R. (2016). Production pathways for renewable jet fuel: a review of commercialization status and future prospects. Biofuels, Biopruducts and Biorefining.

[97] Pearlson M., Wollersheim C., Hileman J.A. (2013). A techno-economic review of hydroprocessed renewable esters and fatty acids for jet fuel production. Biofuels, Bioproducts and Biorefining.

[98] Tao L., Milbrandt A., Zhang Y., Wang W.-C. (2017). Techno-economic and resource analysis of hydroprocessed renewable jet fuel. Biotechnol. Biofuels.

[99] Wang W.C., Tao L. (2016). Bio-jet fuel conversion technologies. Renew. Sustain. Energy Rev..

[100] Wei H., Liu W., Chen X., Yang Q., Li J., Chen H. (2019). Renewable bio-jet fuel production for aviation: a review. Fuel.

[101] Blakey S., Rye L., Wilson C.W. (2011). Aviation gas turbine alternative fuels: a review. Proc. Combust. Inst..

[102] Chiaramonti D. (2019). Sustainable aviation fuels: the challenge of decarbonization. Energy Proc..

[103] Kousoulidou M., Lonza L. (2016). Biofuels in aviation: fuel demand and CO2 emissions evolution in Europe toward 2030. Transport. Res. Transport Environ..

[104] Lee D.S., Fahey D.W., Forster P.M., Newton P.J., Wit R.C.N., Lim L.L., Owen L.B., Sausen R. (2009). Aviation and global climate change in the 21st century. Atmos. Environ..

[105] Lee D.S., Pitari G., Grewe V., Gierens K., Penner J.E., Petzold A., Prather M.J., Schumann U., Bais A., Berntsen T., Iachetti D., Lim L.L., Sausen R. (2010). Transport impacts on atmosphere and climate: aviation. Atmos. Environ..

[106] Staples M.D., Malina R., Suresh P., Hileman J.I., Barrett S.R.H. (2018). Aviation CO 2 emissions reductions from the use of alternative jet fuels. Energy Pol..

[107] Wang M., Dewil R., Maniatis K., Wheldoon J., Tan T., Baeyens J., Fang Y. (2019). Biomass-derived aviation fuels: challenges and perspective. Prog. Energy Combust. Sci..

[108] Colket M., Heyne J., Rumizen M., Gupta M., Edwards T., Roquemore W.M., Andac G., Boehm R., Lovett J., Williams R., Condevaux J., Turner D., Rizk N., Tishkoff J., Li C., Moder J., Friend D., Sankaran V. (2017). Overview of the national jet fuels combustion program. AIAA J..

[109] Lee D.S., Fahey D.W., Skowron A., Allen M.R., Burkhardt U., Chen Q., Doherty S.J., Freeman S., Forster P.M., Fuglestvedt J., GettelmanR. R A., De León, Lim L.L., Lund M.T., Millar R.J., Owen B., Penner J.E., Pitari G., Prather M.J., Sausen R., Wilcox L.J. (2020). The contribution of global aviation to anthropogenic climate forcing for 2000 to 2018. Atmos. Environ..

[110] Prussi M., Lee U., Wang M., Malina R., Valin H., Taheripour F., Velarde C., Staples M.D., Lonza L., Hileman J.I. (2021). CORSIA: the first internationally adopted approach to calculate life-cycle GHG emissions for aviation fuels. Renew. Sustain. Energy Rev..

[111] Schmidt P., Batteiger V., Roth A., Weindorf W., Raksha T. (2018). T. Power -to- liquids as renewable fuel option for aviation: a review. Chem. Ing. Tech..

[112] Voigt C., Kleine J., Sauer D., Moore R.H., R, Bräuer T., Le Clercq P., Kaufmann S., Scheibe M., Jurkat-Witschas T., Aigner M., Bauder U., Boose Y., Borrman S., Crosbie E., Diskin G.S., DiGangi J., Hahn C., Heckl C., Huber F., Nowak J.B., Rapp M., Rauch B., Robinson C., Schripp T., Shook M., Winstead E., Ziemba L., Schlager H., Anderson B.E. (2021). Cleaner burning aviation fuels can reduce contrail cloudiness. Communications Earth & Environment.

[113] Deloitte (2023). https://www2.deloitte.com/content/dam/Deloitte/us/Documents/consumer-business/airline-tech-trends-2023.pdf.

[114] Gao Z., Mavris D.N. (2022). Statistics and machine learning in aviation environmental impact analysis: a survey of recent progress. Aerospace.

